# Virtual Screening, Identification and In Vitro Testing of Novel Inhibitors of O-Acetyl-L-Serine Sulfhydrylase of *Entamoeba histolytica*

**DOI:** 10.1371/journal.pone.0030305

**Published:** 2012-02-15

**Authors:** Isha Nagpal, Isha Raj, Naidu Subbarao, Samudrala Gourinath

**Affiliations:** 1 School of Life Sciences, Jawaharlal Nehru University, New Delhi, India; 2 School of Computational and Integrative Sciences, Jawaharlal Nehru University, New Delhi, India; Stanford University, United States of America

## Abstract

The explosive epidemicity of amoebiasis caused by the facultative gastrointestinal protozoan parasite *Entamoeba histolytica* is a major public health problem in developing countries. Multidrug resistance and side effects of various available antiamoebic drugs necessitate the design of novel antiamobeic agents. The cysteine biosynthetic pathway is the critical target for drug design due to its significance in the growth, survival and other cellular activities of *E. histolytica*. Here, we have screened 0.15 million natural compounds from the ZINC database against the active site of the EhOASS enzyme (PDB ID. 3BM5, 2PQM), whose structure we previously determined to 2.4 Å and 1.86 Å resolution. For this purpose, the incremental construction algorithm of GLIDE and the genetic algorithm of GOLD were used. We analyzed docking results for top ranking compounds using a consensus scoring function of X-Score to calculate the binding affinity and using ligplot to measure protein-ligand interactions. Fifteen compounds that possess good inhibitory activity against EhOASS active site were identified that may act as potential high affinity inhibitors. *In vitro* screening of a few commercially available compounds established their biological activity. The first ranked compound ZINC08931589 had a binding affinity of ∼8.05 µM and inhibited about 73% activity at 0.1 mM concentration, indicating good correlation between *in silico* prediction and *in vitro* inhibition studies. This compound is thus a good starting point for further development of strong inhibitors.

## Introduction

*Entamoeba histolytica,* an anaerobic protozoan parasite, causes amoebic colitis (also called amoebic dysentery) and amoebic abscesses, and infects the liver, kidney and brain. [1]. These infections are the third leading cause of death among the parasitic diseases, surpassing malaria and schistosomiasis [2].

According to WHO, an estimated population of more than 280 million people are infected by *E. histolytica*, causing 2.5 million deaths annually [3]. Several anti-amoebic drugs are currently available, of which the most common ones are derivatives of 5-nitromidazole including metronidazole and tinidazole. Some non-imidazoles drugs such as nitazoxanide, paramomycin and niridazole have also been found to be effective against *E. histolytica*. However, these anti-amoebic drugs are less effective against the cyst of *E histolytica than the trophozoite*. They also show various adverse side effects, with nausea, vomiting, diarrhoea, and hypersensitivity amongst the most common symptoms [4]. Neurological side effects of these drugs include dizziness, vertigo, and encephalopathy. Metronidazole can also cross the placental barriers, thus limiting its use. Moreover, cases of drug resistance in *E histolytica* against 5-nitroimidazole derivatives have been indicated by decreased uptake of metronidazole, and alteration of the pyruvate-oxidizing metabolic pathway [5]. Thus, there is a serious need for a new class of drugs that is more effective and that produces fewer or no side effects.

Being parasitic, *E histolytica* exhibits a complex life cycle which features an antigenically diverse stage (a typical characteristic of protozoan parasites) in order to evade the host's immune system [1]. Other key factors that enhance the virulence of *E histolytica,* include complement resistance, ROS and NOS scavenging potential, and oxygen reduction capability. Oxygen is toxic for the anaerobic protozoans, which damages parasite, and it also destroys oxygen sensitive metabolic enzymes such as pyruvate ferrodoxin oxidoreductase (PFOR), a key enzyme in the anaerobic glycoltic pathway [6]. Cysteine plays a pivotal role in detoxifying the effect of ROS and oxygen and it is crucial for survival of the organism. Cysteine is also important for attachment and growth of trophozites of *E. histolytica*. [7]. The major route of cysteine biosynthesis in this organism is the condensation of O-Acetyserine with the sulphide by the *de novo* cysteine biosynthetic pathway involving two key enzymes: O-Acetyl-L-Serine Sulfhydrylase (EhOASS) and Serine acetyl transferase (EhSAT), which can act as promising targets for inhibiting the growth of *E.histolytica*. Both enzymes are crucial for the cysteine biosynthetic pathway and their structures have been determined in our laboratory [8], [9]. The absence of these enzymes in humans and its essential role in *E.histolytica* suggest them to be the best targets for designing antiamoebic drugs.

Here we report the *in silico* screening of natural compounds and preliminary biochemical investigations of inhibitor screening against EhOASS. Two of the four commercially available compounds showed micromolar binding affinity and one molecule inhibits about 73% of EhOASS activity at 100 µM concentration.

### Drug Target Protein: O-acetylserine Sulfhydrylase of *Entamoeba histolytica*

The EhOASS polypeptide folds into two subunits. Each subunit comprises an α/β domain, and each domain includes a relatively small mobile N-terminal domain (residues 57–164) and a large rigid C-terminal domain (residues 1–56 & 165–336). The active site is located in the middle of these two domains and the cofactor PLP (Pyridoxal5′phosphate) is covalently linked to Lys-58, which is conserved in all OASS structures and is also positioned in between these two domains. Other highly conserved residues such as Gly236, Ser280 and Pro307 interact with the aromatic ring of PLP [10].

### Cysteine Biosynthetic Pathway

OASS catalyses the final step of cysteine biosynthesis, which is the PLP (Pyridoxal 5′phosphate) dependent conversion of O-Acetyl serine into cysteine. O-Acetyl serine (OAS), generated from serine and acetyl-CoA by SAT, reacts with the sulphide to produce L-Cysteine. OASS catalyses this reaction by transferring sulphide group to O-Acetyl serine (OAS) to form Cysteine [5].

**Figure 1:**
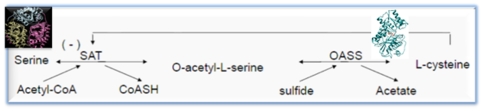
Regulation of cysteine biosynthetic pathway through feedback inhibition of SAT by cysteine.

The two key enzymes (i.e. OASS and SAT) of the cysteine biosynthetic pathway are regulated through feedback inhibition of SAT by cysteine as shown in Figure 1. Also, the C-terminal end of SAT combines with and blocks the active site of OASS upon forming the cysteine synthase complex. The C-terminal end of OASS from *E. histolytica*, however, is unique since it has small side chains unfavourable for making strong interactions with OASS [8]. Since there is a need to design novel inhibitors to target the active site of EhOASS, which is a major regulator in the cysteine biosynthetic pathway of *E. histolytica,* We have used an *in silico* approach, and have screened a large library of natural molecules against this target enzyme. The screening of the library was performed using the GLIDE GScore program in the Schrodinger software package (Glide, v8.0, 2008) [11]. From our findings, we selected the best ranking lead compounds and cross validated them with GOLD [12], Finally post docking analysis was performed using Xscore [13] which calculates the binding affinity (hydrogen and hydrophobic interactions) between the docked inhibitors and target protein.

## Materials and Methods

### Protein and Grid Preparation

The crystal structure of O-acetyl serine sulfhydrylase in complex with cysteine determined by our group to a resolution of 2.4 Å (PDB-ID 3BM5) was retrieved from the Protein Data Bank [9]. We also used the native structure determined at 1.86 Å (PDB-ID 2PQM) as a reference. EhOASS has two subunits, an N and a C-terminal domain. PLP, which is crosslinked to Lys 58 is located in the middle of these two domains, forming the centre of the active site. Protein is prepared using the Schrodinger protein preparation wizard by removal of water and sulphate molecules, and addition of hydrogen atoms, followed by minimization and optimization using OPLS2005 force field in the premin option of Schrodinger Glide. The shape and properties of the receptor are represented on a grid by several different sets of fields that provide progressively more accurate scoring of the ligand poses. We have generated the grid that covers all the catalytic residues with PLP-Lys-58 in the cavity.

**Figure 2:**
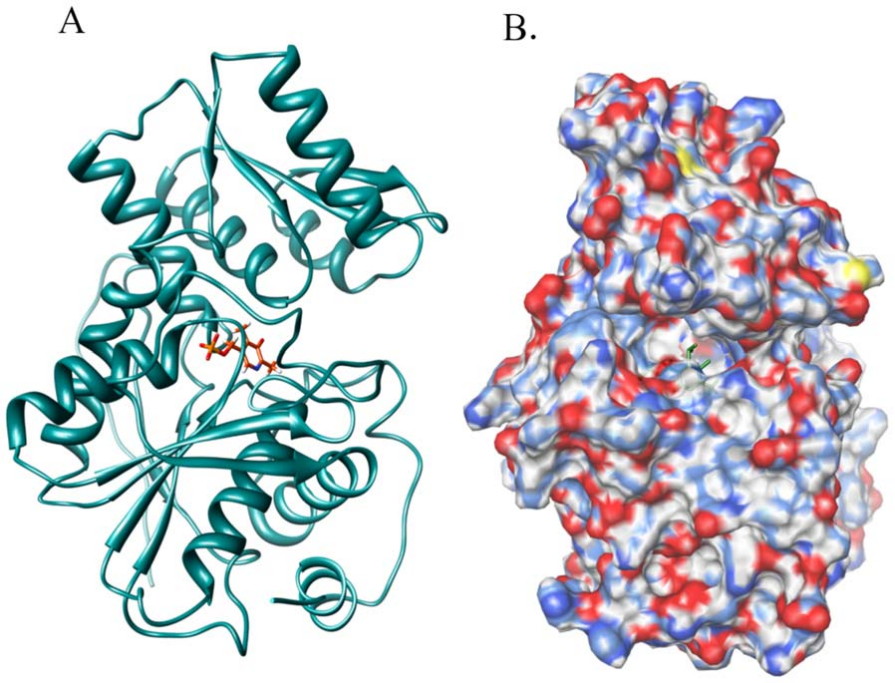
Active site of EhOASS with reaction centre PLP (shown in blue) located at the middle of N and C terminal domain. A) EhOASS (in ribbon) and reaction centre PLP (in sticks). B) EhOASS with electrostatic surface view and reaction centre PLP deep-seated (in sticks) is in binding pocket.

The list of active site residues that are selected for grid generation in the protein are V57,S84,T85, S86, G87, N88, T89, G90, M112, S113, R116, Q159, F160, G192, T193, H232, G233,I234,Q235, G236, I237, G238, A239, F241,Y313, T316, and PLP-LYS-58 (Figure 2).

### Ligand Library Preparation

The ligand library including 0.15 million natural compounds was extracted from the ZINC database (http://zinc.docking.org/). These molecules were then prepared in Schrodinger ligprep wizard using the Lipinski filter. The shortlisted ligands were subjected to further predocking preparations where hydrogens were added followed by minimization and optimization in OPLS_2005 force field. Finally, 10 conformations for each ligand were generated, and ready for docking.

### Docking of molecules to EhOASS structure using GLIDE and Cross validation using GOLD

After preparing the ligand library and protein, and defining the grid corresponding to the active site of the protein, docking procedures were carried out. Our chosen software GLIDE uses Systematic and Simulation method for searching the poses and ligand flexibility. In a systematic method, it uses incremental construction for searching, and its output GScore is an empirical scoring function which is a combination of various parameters [11] The GScore is calculated in Kcal/mol as:
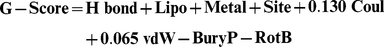
Where: Hbond = Hydrogen bonds, Lipo = hydrophobic interactions, Metal = metal-binding term, Site = polar interactions in the binding site, vdW = Vander-Waals forces, Coul = columbic forces, Bury P = penalty for buried polar group, RotB = freezing rotable bonds.

Library of Natural ligands were subjected to glide docking. Since each ligand has 10 stereoisomers or conformations, each conformation was first screened through the high throughput virtual screening (HTVS) module of GLIDE, from which the top 10% were subjected to the standard precision (SP) module. The top 1,000 outcomes from SP were subjected to the extra precision (XP) module for detailed docking. Finally, we selected the top 10 ranked ligands according to GLIDE and docked them again using GOLD docking software to obtain consistent and improved results. GOLD v4.0 [12] is an automated ligand-docking program that uses a genetic algorithm to explore the full range of ligand conformational flexibility, namely full acyclic ligand flexibility and partial cyclic ligand flexibility, with partial flexibility of the protein in the neighbourhood of the protein active site, and satisfies the fundamental requirement that the binding of ligand must displace loosely bound water. In GOLD docking, fifty independent docking runs were performed for each molecule with default parameters.

### Post Docking Analysis

**Figure 3:**
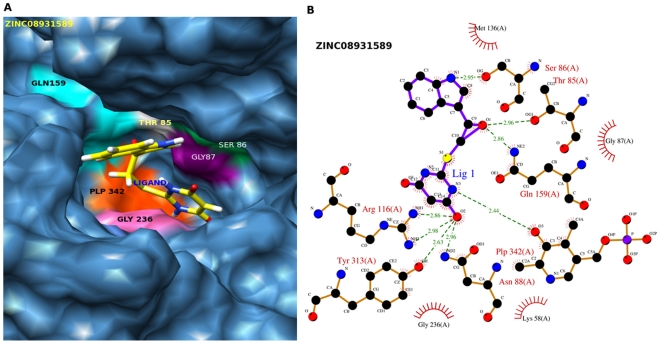
Post-docking interactions between active site residues of protein with ligand (ZINC08931589). (A)The protein is depicted in surface view and ligand ZINC08931589 as stick in the binding pocket. (B) Schematic drawing of types of interactions of the ligands generated using Ligplot.

**Figure 4:**
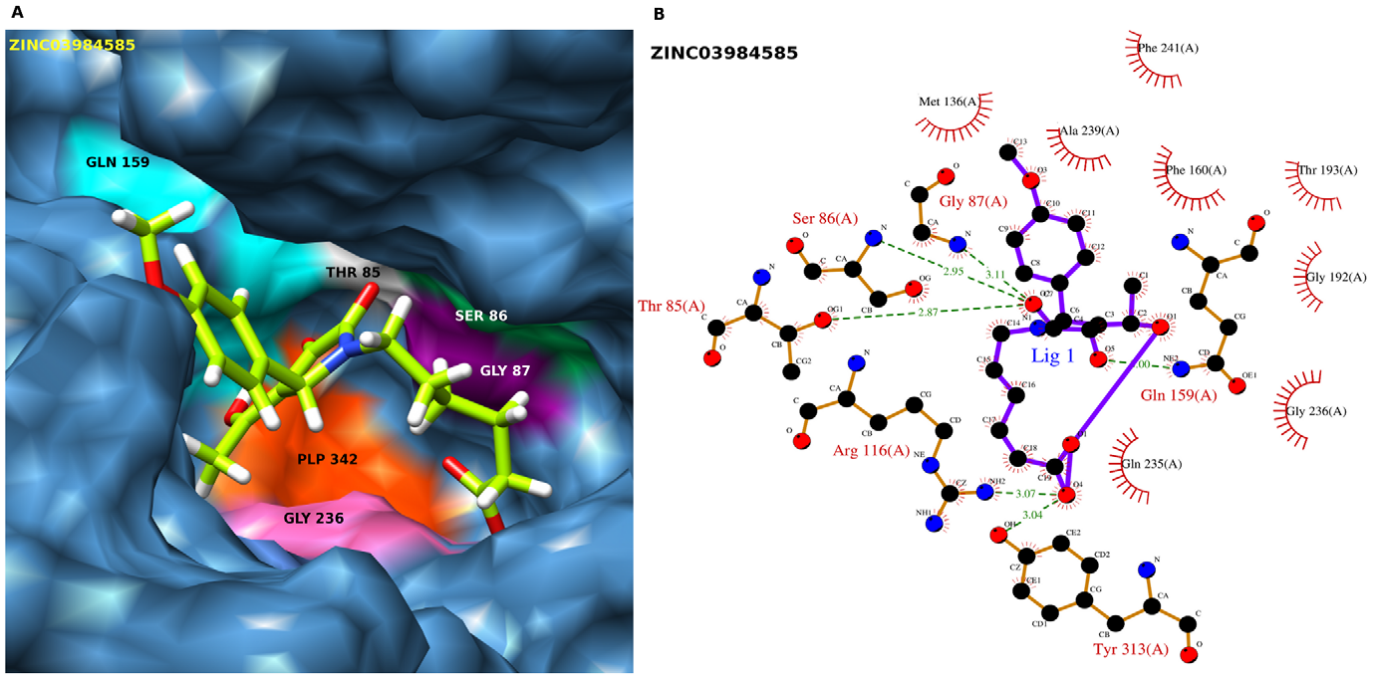
Post-docking interactions between active site residues of protein with ligand (ZINC03984585). (A) The protein is depicted in surface view and ligand ZINC03984585 as stick in the binding pocket. (B) Schematic drawing of types of interactions of the ligands generated using Ligplot.

A molecule was ranked relatively high if it scores well with these two different methods (or scoring functions). These methods have different search algorithms and scoring functions. Hence, it was not possible to compare the fitness scores of GOLD and GLIDE directly. For comparison and validation of docking result we used X-Score v1.2.1, [13] a consensus scoring function. X-Score calculates the negative logarithm of the dissociation constant of the ligand to the protein, −log Kd, as the average of three scoring functions (HPScore, HMScore and HSScore), and predicts the binding energy (Kcal/mol) of the ligand. X-Score was reported to have an accuracy of ±2.2 Kcal/mol relative to the actual binding energies. For analysing the interactions of docked protein-ligand complexes, the Ligplot programme [14] was used to check the hydrogen bond and hydrophobic interactions between receptor and ligand atoms within a range of 5 Å. Also PyMOL (V-1.3) [15] and Chimera (V-1.4.1) [16] were used to visualize the interactions and to prepare figures for top ranked molecules (Figure 3 & 4).

### Validation through biochemical assays: inhibition and binding studies

We were able to procure four of the shortlisted natural inhibitor compounds. They were tested for their effect on enzyme activity and interaction with the active site [8]. The enzyme EhOASS was expressed and purified as described previously [10]. O-Acetyl-L-serine, DTNB, TCEP and HEPES were purchased from Sigma. The sulfhydrylase activity was monitored using 5-thio (2-nitrobenzoate) (TNB) as an alternative substrate. The disappearance of TNB was monitored continuously at 412 nm using the UV-visible spectrophotometer Ultrospec 21000pro. The Km of EhOASS for its substrate OAS is 0.5 mM; hence an activity check was carried out with 100 µM inhibitor in the presence of 0.5 mM OAS to analyse the non-competitive effect and also at 100 µM OAS to analyse the effect of inhibitor at competitive levels of substrate. The non-competitive assay contained the following in final concentrations: 100 mM HEPES, pH 7.0, 0.5 mM OAS, 0.05 mM TNB and 25 µg of EhOASS. The competitive assay contained 100 µM OAS instead of 0.5 mM OAS. Decrease in enzyme activity was monitored over a fixed interval of time in the presence of 100 µM inhibitor. The absorbance pattern of the standard reaction was compared with those with the inhibitor, and percentage decrease in activity was calculated using the following equation: 100-[(decrease in absorbance for reactions with inhibitors/decrease in absorbance for standard reaction) ×100].

**Figure 5:**
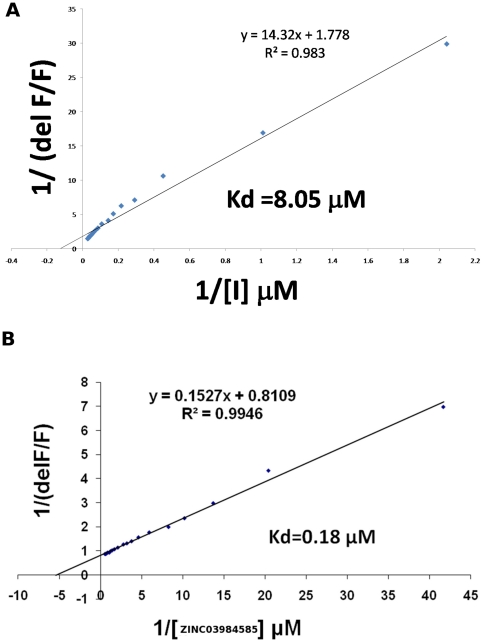
The plot between ΔF/F vs. inhibitor concentration follows a typical hyperbolic similar to Michaelis-Menton curve, the plot between 1/ΔF/F vs. inhibitor concentration (the reciprocal plot) follows that of a Lineweaver-Burk plot, the Kd values of inhibitor A) ZINC08931589 and B) ZINC03984585 were determined from these plots.

The inhibitors that showed a reasonable decrease in activity were studied further, to determine binding affinity. The fluorophor PLP in the active site of OASS absorbs at 412 nm and emits at 510 nm. Titration of EhOASS with the active site-binding inhibitors leads to an increase in emission at 510 nm Fluorescence measurements were carried out using a Cary400 Scan fluorospectrophotometer (Varian Inc.). Emission spectra were recorded for a solution containing 250 µg/ml EhOASS and 100 mM HEPES (pH 7) upon excitation at 412 nm (slitex = 5 nm, slitem = 10 nm). Fluorescence peak was measured in absence of the inhibitor (F). Difference in fluorescence arising due to addition of inhibitor, ΔF, was also measured and corrected for dilution. The plot of inhibitor concentration and ΔF/F follows a typical hyperbolic Michaelis-Menten-like curve. Kd was determined by plotting 1/(ΔF/F) versus 1/inhibitor concentration, as like a Lineweaver-Burk plot (Figure 5A,B).

## Results and Discussion

O-Acetyl Serine Sulfhydrylase (OASS) is a promising and established drug target in *Entamoeba histolytica.* This organism causes amoebiasis for which there are few and poorly effective drugs. It has PLP (pyridoxal phosphate) as a cofactor and plays a crucial role in the cysteine biosynthetic pathway. Using the GLIDE and GOLD docking programs, approximately 0.15 million natural compounds were screened from the ZINC database against OASS to identify potential inhibitor molecule, as explained in the Methods section.

The top ten ranking natural compounds based on GLIDE scores are listed in Table 1. The GLIDE scores and Xscores of these compounds have ranges of −13.01 to −10.91 Kcal/mol and −9.07 to −7.01 Kcal/mol, respectively. The top ranking molecule (**ZINC08931589,** 4-hydroxy-2-[2-s(1H-indol-3-yl)-2-oxoethyl]sulfanyl-1H-pyrimidin-6-one) has a Glide score of −13.01 Kcal/mol and Xscore of −7.76 Kcal/mol with 8 hydrogen bonds and 4 hydrophobic contacts (Table 1 & 2). Conserved residues involved in hydrogen bonding interaction include T85, S86, Q159, G87, R116, and G236. (except ZINC04349228).

**Table 1:** Top ranking ligands (Natural compounds from Zinc database) after virtual screening against EhOASS using GLIDE and GOLD docking programs.

S.No	Zinc-Id	Ligand Iupac Name (Molecular structures can be seen in Figure S1)	GlideScore (Kcal/mol)	Glide X-Score (Kcal/mol)	Gold Score	Gold Xscore
1	ZINC08931589	4-hydroxy-2-[2-(1H-indol-3-yl)-2-oxoethyl]sulfanyl-1H-pyrimidin-6-one.	−13.01	−7.76	56.6	−7.57
2	ZINC04349228	2-(3,4-dihydroxyphenyl)-3,5,7-trihydroxy8[(2S,3R,4R,5S,6R)-3,4,5-trihydroxy-6-(hydroxymethyl)oxan-2-yl]oxychromen-4-one.	−12.64	−8.5	45.71	−8.64
3	ZINC12405024	(2R,3S,4R,5R)-2,3,5,6-tetrahydroxy-4-[(2S,3R,4S,5R,6R)-3,4,5-trihydroxy-6-(hydroxymethyl)oxan-2-yl]oxyhexanoate.	−12.1	−7.01	46	−6.79
4	ZINC13409670	3-((S)-3,4-Dihydroxy-1-methoxy-2-oxo-pentyl)-2,6,8,9-tetrahydroxy-7-methyl-3,4-dihydro-2H-anthracen-1-one.	−11.72	−8.88	45.82	−8.41
5	ZINC04349223	2-(3,4-dihydroxyphenyl)-3,5,7-trihydroxy-8-[3,4,5-trihydroxy-6-(hydroxymethyl)oxan-2-yl]oxychromen-4-one.	−11.7	−8.93	69.29	−8.1
6	ZINC03984585	3-[3-acetyl-4-hydroxy-2-(4-methoxyphenyl)-5-oxo-2,5-dihydro-1H-pyrrol-1-yl]propanoic acid.	−11.49	−7.79	59.52	−7.78
7	ZINC04349749	5,7-dihydroxy-2-(4-hydroxyphenyl)-8-[(2S,3R,5S)-3,4, 5-trihydroxy-6-(hydroxymethyl)oxan-2-yl]chromen-4-one	−11.37	−9.07	56.77	−9.09
8	ZINC13409674	(3R)-3-[(1S,3S,4S)-3,4-dihydroxy-1-methoxy-2-oxopentyl]-8,9-dihydroxy-7-methyl-3,4-dihydro-2H-anthracen-1-one.	−11.3	−8.89	39.92	−8.52
9	ZINC03984470	6-[3-acetyl-2-(4-bromophenyl)-4-hydroxy-5-oxo-2,5-dihydro-1H-pyrrol-1-yl]hexanoic acid.	−11.02	−8.02	59.79	−8.09
10	ZINC08740334	(2S,3R,4S,5R,6R)-2-[(2R,3S,4R,5R)-6-(3,4-dimethylanilino)-4,5-dihydroxy-2-(hydroxymethyl)oxan-3-yl]oxy-6(hydroxymethyl) oxane-3,4,5-triol.	−10.91	−8.6	45.5	−8.11

**Table 2:** Calculated Hydrogen and Hydrophobic interactions for the top ten best ranking ligands based on Glide score and ligplot.

S.no:	ZINC ID	Hydrogen bond (≤3.6 Å)	Hydrophobic Interactions
1	ZINC08931589	**T85, S86, Q159,** PLP342, N88, Y313, **R116,G236**	G87, M136, G236, K58
2	ZINC04349228	**T85, G87**, **Q159(3),** K58	PLP342,I237,G236,G192,H232,Q235,A239,M136, F241, S84, F160
3	ZINC12405024	**T85,S86,** **Q159,G236,** S113, N88, T89, K58, I237	R116, M112, Q235, A239, G238, G192, PLP342, G87
4	ZINC13409670	**T85, S86(2), G236,** S84(2), Q159, S113	I140, F160, M136, F241, Q235, I237, G87
5	ZINC04349223	**T85, S86(2), G87, Q159(2),** K58	S84, I140, A239, G238, G236, PLP342,M136, F160
6	ZINC03984585	**G87, S86, T85, R116,** **Q159,** Y313	M136, A239, F160, T193, G129, G236, Q235, F241
7	ZINC04349749	**T85**, **Q159,** K58	I140, F160, F241, M136, Q235, R116, G87
8	ZINC13409674	**T85,** **G236,** S84(2), **Q159,** **S86,** S113	I240, F160, F241, M36, Q235, R116, G87
9	ZINC03984470	**T85,S86, G87, R116**, **Q159,** Y313	A239, G192, F160, G236, T193, Q235.
10	ZINC08740334	**T85,S86,G87,R116,** **Q159,G236(2),** Y313	M136, F160, G87, F241, A239, Q235, I237, G238, M112

Those compounds predicted to bind EhOASS strongly according to GLIDE are also predicted to bind strongly according to GOLD. For each compound docked using the GLIDE and GOLD programs, the Xscore (consensus scoring function) program was used to calculate binding energies and listed in Table 1 along with IUPAC name of the compounds, their respective ZINC ID's and interacting residues (hydrogen bonding and hydrophobic contacts). Interactions (hydrogen and hydrophobic) for the top ten best ranking ligands based on glide score and ligplot are listed in Table 2. The two ligands are shown in the binding site of EhOASS and LIGPLOT diagrams are drawn showing hydrogen bonding and hydrophobic contacts between the docked poses of protein and ligands. (Figure 3 &4).

The ligand, **ZINC08931589** is a ring structure having a molecular weight of 301. 327 amu and a logP value of 2.81. It showed the highest Glide score, −13.01 Kcal/mol, and Gold score, 56.6. X-score was also reasonably good (−7.7 Kcal/mol) for this compound. In the docked model, there are 8 hydrogen bond interactions and 4 hydrophobic interactions between the ligand and the protein (Figure 3). The zeta nitrogen of the basic polar amino acid Lys-58 forms a hydrogen bond with the atom S1 of the ligand. Similarly, atoms NH1 and NH2 of the basic polar amino acid Arg116 forms hydrogen bonds with the atom O2 of this ligand. Meanwhile the atom O2 _at_ γ-1 position, makes hydrogen bonds with the atom O1 of the ligand. All the hydrogen bonds between this ligand and the protein are made with the active site of the protein, as described. PLP-Lys58 makes a 2.4 Å-long hydrogen bond with the N3 atom of the ligand.

The ligand **ZINC03984585** has a molecular weight of 360.386 amu, logP of 1.63 with a GLIDE score of −11.49 Kcal/mol and GOLD score of 59.52 (Table 1). In our study, this 6^th^ ranked compound forms 6 hydrogen bonds and 8 hydrophobic interactions with the receptor (Table 2). Hydrogen bonds are formed by Thr85, Ser86, Arg116 and Gln159, as is the case for ZINC08931589. In addition, neutral nonpolar amino acids Gly87, Gly192, Gln235, Gly236, and Ala239 are also involved in some hydrophobic interactions. (Figure 4).

From the biochemical studies, the compound ranked first showed about 73% decrease in activity, while the compound ZINC03984585 ranked 6^th^ showed 21% decrease in activity at Km level of substrate. At equal concentration of substrate and inhibitor, the percent decrease in activity was found to be greater (Table 3). There is thus a a good correlation between our prediction and experimental results. The binding affinity for compound ZINC08931589 is approximately 8 µM whereas that for compound ZINC03984585 is 0.18 µM (Figure 5A,B). The compound ZINC03984585 shows better binding affinity but less inhibition. This difference is not an aberration as compounds showing better Kd do not necessarily have a better efficacy [17]. Hence, though ZINC08931589 has a lower binding affinity, it has a higher efficacy at inhibiting the enzyme.

**Table 3:** Inhibition studies of EhOASS.

S.no	Inhibitor	% inhibition (0. 5 mM OAS)	%inhibition (100 µM OAS)
1	ZINC08931589	73. 59±3. 2	-
2	ZINC12405024	2. 60±0. 2	8. 77±0. 6
3	ZINC08740334	11. 35±1. 6	16. 66±0. 7
4	ZINC03984585	20. 99±1. 3	33. 03±0. 2

The concentration of the inhibitor was kept at 100 µM and the readings were taken under two substrate concentrations; 0.5 mM (Km of the substrate) and 100 µM (for competitive inhibition, inhibitor and substrate were used same concentration).

### Conclusion

There is an urgent need to design and develop novel antiamobeic agents due to multidrug resistance and side effects of various available antiamoebic drugs. The EhOASS enzyme of the cysteine biosynthetic pathway is a potential drug target in *E.histolytica*. We have identified few novel inhibitors by *in silico* screening of a natural library of compounds from the ZINC database using GLIDE and GOLD docking programs. Our docking results for the top ten candidates, which are discussed in the paper, are supported by post docking analysis and indicate strong binding affinity for EhOASS. A few commercially available compounds have been procured and *in vitro* testing has been carried out for biological activity. Four of the six compounds have shown biological activity when tested experimentally and the results correlated well with *in silico* studies. The top ranking compound inhibited the activity of the enzyme by almost 73% at 100 µM concentration. These results validate our docking studies. These compounds may act as lead molecules for high affinity inhibitory molecule development, which could be potential drug molecules against *E. histolytica.*

## Supporting Information

Figure S1 **The molecular structure of top ranking inhibitors (Natural compounds from zinc database) after virtual screening against EhOASS using GLIDE and GOLD docking programs as listed in** **Table 1** **.**
(TIF)
