# Supplementary figures and images for: Virtual Screening, Identification and In Vitro Testing of Novel Inhibitors of O-Acetyl-L-Serine Sulfhydrylase of Entamoeba histolytica
Source: PLoS One. 2012 Feb 15;7(2):e30305. doi: 10.1371/journal.pone.0030305 (PMC3280239; doi:10.1371/journal.pone.0030305)

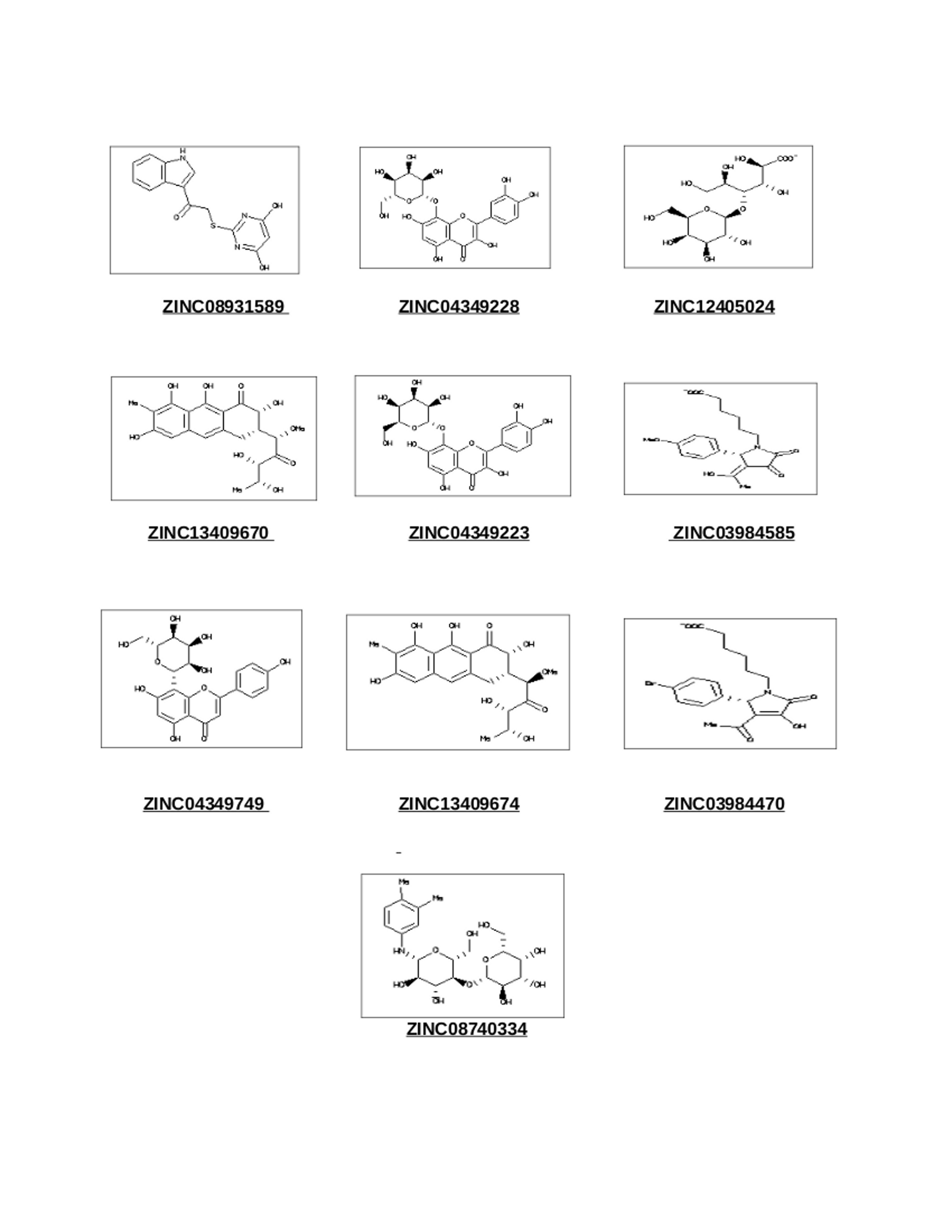

Supplement: Figure S1 — The molecular structure of top ranking inhibitors (Natural compounds from zinc database) after virtual screening against EhOASS using GLIDE and GOLD docking programs as listed in Table 1 . (TIF) [file pone.0030305.s001.tif]
